# Neuroinflammation impairs adaptive structural plasticity of dendritic spines in a preclinical model of Alzheimer’s disease

**DOI:** 10.1007/s00401-015-1527-8

**Published:** 2016-01-02

**Authors:** Chengyu Zou, Yuan Shi, Jasmin Ohli, Ulrich Schüller, Mario M. Dorostkar, Jochen Herms

**Affiliations:** Department for Translational Brain Research, German Center for Neurodegenerative Diseases (DZNE), Munich, Germany; Center for Neuropathology and Prion Research, Ludwig-Maximilians-University, Munich, Germany; Munich Cluster of Systems Neurology (SyNergy), Ludwig-Maximilians-University Munich, Schillerstraße 44, 80336 Munich, Germany; Graduate School of Systemic Neuroscience, Ludwig-Maximilians-University, Munich, Germany

**Keywords:** Preclinical AD, APPswe/PS1deltaE9 mice, Dendritic spines, Structural plasticity, Neuroinflammation

## Abstract

To successfully treat Alzheimer’s disease (AD), pathophysiological events in preclinical stages need to be identified. Preclinical AD refers to the stages that exhibit amyloid deposition in the brain but have normal cognitive function, which are replicated in young adult APPswe/PS1deltaE9 (deltaE9) mice. By long-term in vivo two-photon microscopy, we demonstrate impaired adaptive spine plasticity in these transgenic mice illustrated by their failure to increase dendritic spine density and form novel neural connections when housed in enriched environment (EE). Decrease of amyloid plaques by reducing BACE1 activity restores the gain of spine density upon EE in deltaE9 mice, but not the remodeling of neural networks. On the other hand, anti-inflammatory treatment with pioglitazone or interleukin 1 receptor antagonist in deltaE9 mice successfully rescues the impairments in increasing spine density and remodeling of neural networks during EE. Our data suggest that neuroinflammation disrupts experience-dependent structural plasticity of dendritic spines in preclinical stages of AD.

**Electronic supplementary material:**

The online version of this article (doi:10.1007/s00401-015-1527-8) contains supplementary material, which is available to authorized users.

## Introduction

As the most prevalent cause of dementia, Alzheimer’s disease (AD) is characterized by progressive cognitive deficits, amyloid plaques, neurofibrillary tangles (NFTs) and neuronal loss, yet it still lacks an effective cure at the present time [21, 40]. The failure to develop successful pharmacotherapy may, at least partially, be ascribed to the long pathophysiological process, which starts many years before the stage of symptomatic AD [43]. Therefore, much earlier intervention in the asymptomatic or preclinical stages may be required to successfully treat AD [35, 45].

Preclinical AD has been recently defined as the stages when amyloid is being deposited in the brain, but before the onset of any cognitive impairment [44]. Subjects in the preclinical stages are at risk for future cognitive decline [51]. Indeed, the lag between the appearance of amyloid plaques and detectable impairment in cognition is more than a decade [37, 44]. Growing evidence supports the notion that amyloid deposition disrupts functional networks in the brain of cognitively normal elderly [17, 32, 42, 46]. To have a better chance of curing AD, it is therefore crucial to identify pathophysiological events occurring in preclinical stages that precede dementia during the initial formation of amyloid deposits.

Transgenic mouse models are essential research tools for uncovering AD pathogenesis as well as validating new therapeutic approaches. To recapitulate AD pathology, transgenic mouse models carry familial AD gene mutations in the amyloid precursor protein (APP) and/or presenilins (PS) based on the amyloid hypothesis, which postulates an increased production or decreased removal of the APP proteolytic fragment, amyloid β-protein (Aβ), as the primary cause of AD [16]. The transgenes with APP/PS mutations in mouse models lead to the formation of amyloid plaques and subsequent memory loss, but without the development of NFTs and massive neuronal loss [2]. Although these mice fail to replicate all aspects of the disease, they seem to faithfully imitate pre-dementia stages of AD [1].

Among the APP transgenic mouse models, APPswe/PS1deltaE9 (deltaE9) mice have been widely used. They express APP with the Swedish mutation together with mutant human PS1 with a deletion of exon 9 [23, 39]. Interestingly, in deltaE9 mice, amyloid deposition precedes typical cognitive impairments [22, 50]. Amyloid plaques start to emerge at the age of 4–5 months [4, 14], while the performance of 7-months-old deltaE9 mice is normal in most cognitive tests [27, 34, 50]. The temporal lag between the emergence of amyloid plaques and the onset of dementia consequently provides a critical period to study pathophysiological events related to preclinical AD.

In this study, we used long-term in vivo two-photon microscopy to elucidate the adaptive spine plasticity of deltaE9 mice at young adult age. Our data demonstrated that deltaE9 mice failed to increase spine density and establish novel neural connections when exposed to enriched environment (EE), which was caused by neuroinflammation.

## Materials and methods

### Animals

APPswe/PS1deltaE9 (deltaE9) mice [22] (Jackson Laboratory) were crossed with GPF-M mice [10] (Jackson Laboratory) to obtain double transgenic offspring, which were heterozygous for the corresponding genes (deltaE9 +/− × GFP +/−). GFP positive littermates without APP/PS1 transgenes were used as controls (deltaE9 −/− × GFP +/−). BACE1 knockout mice [5] were also purchased from Jackson Laboratory, and deltaE9 +/− × Bace1 +/− × GFP +/− (deltaE9/Bace +/−) were generated by interbreeding. All transgenic mice were maintained on C57BL/6 background. Female mice at the age of 4–5 months were used. Mice were housed and bred in pathogen-free environment in the animal facility at the Centre for Neuropathology and Prion Research of the Ludwig-Maximilians-University Munich (LMU), with food and water provided ad libidum (21 ± 1 °C, at 12/12 h light/dark cycle). All mice were housed either singly in standard cages (30 × 15 × 20 cm) or in groups in environmentally enriched (EE) cages (80 × 50 × 40 cm) equipped with platforms and variety of toys, which were relocated 3 times per week. Pioglitazone (350 ppm, Actos™) was supplemented into rodent chow. All protocols and procedures involving animals were approved and conducted in accordance with the regulations of LMU and the government of Upper Bavaria (Az. 55.2-1-54-2532-62-12).

### Cranial window implantation and in vivo two-photon imaging

The detailed surgical procedure of cranial window implantation has been described previously [13, 20]. In brief, mice were anesthetized by intraperitoneal injection of ketamine/xylazine (120 and 10 mg/kg, respectively). Subsequently, dexamethasone (6 mg/kg) was injected to prevent development of cerebral edema. A piece of skull above the somatosensory cortex was then removed carefully with a dental drill. The exposed brain was cleaned with sterile saline and covered with a round glass coverslip (*D* = 4 mm). The margin between the glass and skull was sealed with dental cement. Post-surgical mice were subcutaneously injected with carprofen (Pfizer, 4 mg/kg) and cefotaxime (Pharmore, 250 mg/kg). Lentiviruses (LV) encoding IL-1 RA (LV vector was a gift from Dr. van Dam [49]) were intraparenchymally injected into the cortex before implanting the coverslip when specified. LV encoding GFP was used as a control for evaluating the efficiency of IL-1 RA expression. The injection of the LV (200 nl per time at a titer of ~10^8^ infecting units per ml) was performed at 4 different sites in the exposed area of brain at the depth of 700–800 µm. After 4 weeks of recovery period, mice were imaged by using a LSM 7MP microscope (Zeiss) equipped with a 20× objective (NA 1.0; Zeiss). Mice were anesthetized with isoflurane (1 % in 95 % O_2_ and 5 % CO_2_) and placed on a heating pad to keep the body temperature at 37 °C. Apical dendrites originating from GFP-labeled layer V pyramidal neurons were imaged in consecutive sessions (once per week). The imaging session did not last more than 60 min. The unique pattern of blood vessels was used to re-localize the imaged regions in subsequent imaging sessions. GFP was excited by a femtosecond laser (Spectra Physics) at the wavelength of 880 nm. The intensity of laser and settings of data acquisition were kept constant during experiments. To ensure the dendrites were chosen in amyloid plaque-free regions, methoxy-X04 (1 mg/kg) was intraperitoneally injected 24 h before imaging in the first and last time points. Overview images were taken as 424 × 424 × 350 µm^3^ (0.83 µm/pixel). From the central regions of these images, apical dendrites (10–80 µm in depth below the surface of brain) were chosen for analysis to make sure the distance between them and amyloid plaques was more than 100 µm. Higher resolution images (0.138 µm/pixel) were used for counting dendritic spines. For illustration purpose, maximal projection images were deconvolved (AutoQuantX3), with contrast and brightness adjusted.

### Spine analysis

Dendritic spines were analyzed manually in ZEN 2011 (Zeiss) by scrolling through the images in z-stacks. As the limitations of resolution in Z-direction, only laterally protruding spines were counted, as only those could be identified with certainty. In consecutive sessions, a dendritic spine was determined as the same if its location did not change within a range of 0.5 µm along the dendrite. Otherwise, spines that disappeared or emerged compared to the previous imaging session were defined as formed or eliminated, respectively. Spine formation and elimination were normalized into 100 % based on the calculations from the first and second imaging points. The fate of preexisting spines was calculated as the fraction of dendritic spines in the first imaging session that remained stable during the imaging period. Similarly, the fate of new-gained spines was the fraction of formed spines in the first week of EE or matching week of SC that remained stable during the rest of imaging period, indicating how many gained spines incorporated into neural circuits. Transient spines were determined as spines that did not survive more than 1 week and referred to the stability of gained spines in a short-term period.

### Immunochemistry

Following transcardial perfusion with phosphate-buffered saline (PBS) and 4 % paraformaldehyde (PFA), mouse brains were cut into 65 µm thick sections from the somatosensory cortex after being fixed in 4 % PFA overnight. Antibodies against GFAP (Abcam 1:500), Iba1 (Wako 1:500) and beta-amyloid (4G8, BioLegend, 1:500) were used to detect activated astrocytes, microglial, and amyloid beta, respectively. Anti-rabbit Alexa 647 antibody (Invitrogen 1:1000) was used as the secondary antibody. To stain amyloid plaques, sections were incubated with 145 µm methoxy-X04 in PBS for 30 min and then washed with PBS. After mounting on glass coverslips by fluorescence mounting medium (Dako), sections were imaged using LSM 780 confocal microscope (Zeiss). Glial activation, plaque load, or amyloid pathology was quantified as the area with positive staining relative to the cortex area after maximal projection of confocal stacks.

### Western blot

10 % cortical tissues (w/v) were homogenized on ice in lysis buffer with protease inhibitors (Roche), followed by centrifugation at 500 rpm for 1 min. The supernatant was collected and protein concentrations were adjusted by the bicinchoninic acid assay to ensure the same amount of protein being loaded for each sample (80 µg). Samples were mixed with SDS-containing sample buffers and incubated at 100 °C for 20 min. After electrophoresed on 12 % sample gel, proteins were transferred into polyvinylidene difluoride membrane (Millipore). The primary antibodies against IL-1β (Cell signaling), IL-1 RA (Thermo Scientific) and tubulin (Santa Cruz) were used at 1: 1000 concentrations for immunoblotting. Protein bands were quantified by ImageJ.

### Quantitative real-time PCR

For gene expression analysis in brain tissue, RNA extraction was performed using TRIzol (Invitrogen). Random hexamer primers, oligo dTs and Superscript II reverse transcriptase (Invitrogen) was used for generation of cDNA. For quantitative real-time RT-PCR, the LightCycler480 system (Roche) and the corresponding SYBR Green detection format was used. *Beta*-*2*-*microglobulin* (*β2M*) was used as a housekeeper. All analyses were conducted as triplicates. Primers for *β2M* were designed using Primer3 software. Sequences for the primers were as follows: *β2M* forward, 5′-TGTCTTTCAGCAAGGACTGG-3′; *β2M* reverse, 5′-GATGCTGCTTACATGTATCG-3′; *IL*-*1β* forward 5′-GGCTGGACTGTTTCTAATGC-3′; *IL*-*1β* reverse 5′-ATGGTTTCTTGTGACCCTGA-3′ [55].

### Statistics

For statistical analysis and comparison, GraphPad Prism 5 was used. In the longitudinal measurements of spine analysis, extra sum-of-squares *F* test was used when data were fitted with a line using the nonlinear regression. Comparison among groups was performed using one-way ANOVA followed by Newman–Keuls post-test. Two-tailed Student *t* test was used in comparison between two different groups. The numbers of mice were 4–6 per group for in vivo imaging. 8–12 dendrites were imaged in each mouse. The length of each dendrite was 25–35 µm and the number of spines was normalized to the dendritic length. Data are presented as mean ± SEM. *N* refers to the number of mice; *p* < 0.05 was defined as statistically significant (* *p* < 0.05, ** *p* < 0.01).

## Results

### Adaptive structural plasticity of dendritic spines is impaired in deltaE9 mice at the age of 4–5 months

**Fig. 1:**
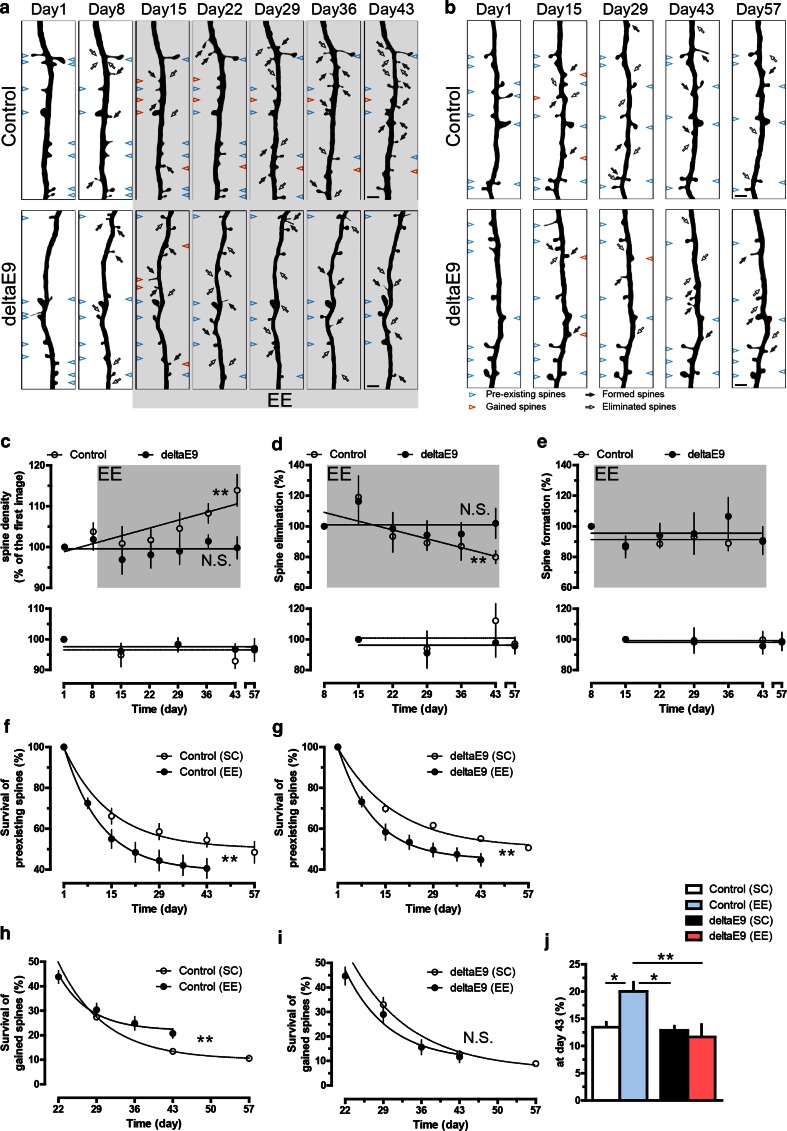
Adaptive plasticity of dendritic spines is impaired in deltaE9 mice. **a**, **b** Two-photon micrographs of GFP-labeled apical dendrites of layer V pyramidal neurons. Mice were housed in standard conditions (SC) and imaged twice in a week apart before being housed in enriched environment (EE) (**a**). In **b**, mice were housed in SC throughout the experiment. *Empty or dark arrows* point to eliminated or formed spines compared to previous imaging session. *Blue arrowheads* mark spines that existed in the first imaging session and were stable over the entire imaging period while *red arrowheads* represent gained spines in the first week of EE or matching period of SC that survived over the rest of imaging period. **c**–**e** Quantifications of relative spine density, fraction of eliminated or formed spines in mice housed under EE (*above*) or SC (*below*). **f**, **g** Fractions of spines from the first imaging session that remained stable during the whole imaging period. **h**, **i** Fractions of gained spines in the first week of EE or matching period of SC that remained stable during the whole imaging period. **j** The data at day 43 from **h** and **j** were compared by one-way ANOVA. *Scale bar* 2 µm

Replicating the preclinical stages of AD [1, 44], 4–5-month-old deltaE9 mice develop amyloid deposits without cognitive decline [4, 14, 27, 34, 50]. In agreement with the normal cognitive state, our previous study observed normal spine density and dynamics on dendrites that were far away from amyloid plaques in deltaE9 mice at this age [58]. To further examine if activity-induced structural spine plasticity on these dendrites is disturbed in preclinical AD, we housed deltaE9 mice at the age of 4–5 months under enriched environment (EE) over 5 weeks and monitored the apical tufts of layer V pyramidal neurons in the somatosensory cortex (Suppl. Figure 1). EE, which provides a spectrum of synaptic inputs and thus leads to adaptive synaptic alterations within the adult brain [30, 31, 38], induced a steady increase of spine density in the control group (Fig. 1a, c). In contrast, EE failed to increase spine density in deltaE9 mice (Fig. 1a, c). Of note, unlike control mice demonstrating gradual decline in dendritic spine elimination upon EE, the rate of spine elimination in deltaE9 mice remained unaltered (Fig. 1d). EE did not change the rate of spine formation in both groups (Fig. 1e). Moreover, during the imaging period, the density and dynamics of dendritic spines remained unchanged, when mice were housed under standard conditions (SC, Fig. 1b, c–e). Thus, EE-induced decrease in spine elimination and subsequent increase in spine density were absent in deltaE9 mice.

To find out how preexisting neural networks react to the stimulation of EE, we tracked the fate of dendritic spines that existed in the first imaging session over the whole period of enrichment. Interestingly, in control and deltaE9 genotypes, less preexisting spines survived when mice were housed under EE (Fig. 1f, g). This indicated a breakdown of the established neural networks in both groups during EE. Furthermore, the fate of spines that were newly formed in EE or SC was also monitored. A higher number of gained spines remained stable during EE in control mice, but not in deltaE9 mice (Fig. 1h–j). Also, a direct comparison between control and deltaE9 mice revealed that the elimination rate of newly gained dendritic spines induced by EE was higher in the AD mouse model (Suppl. Fig. 2). These results suggest the failure of building up novel neural networks induced by EE in deltaE9 group. Collectively, our data imply that the reorganization of neural networks upon EE is impaired in preclinical stages of AD.

### Reduction of BACE1 in deltaE9 mice restores the response with an increase in spine density upon EE

**Fig. 2:**
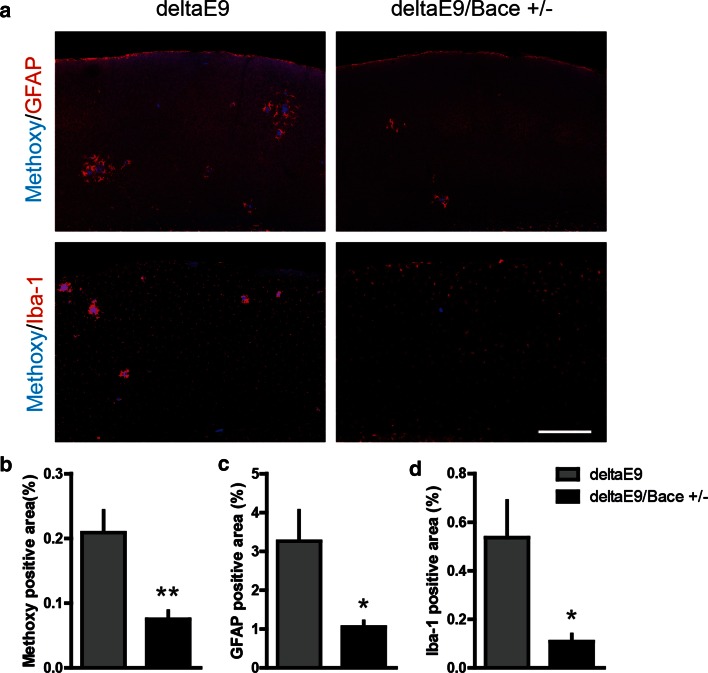
Partial reduction of BACE1 in deltaE9 mice greatly decreases amyloid plaque load and subsequent glial cell activation. **a** Immunohistochemical labeling of amyloid plaques (*blue*) and activated astrocytes (GFAP, *red*) or microglia (Iba-1, *red*) in the cortex. *Scale bar* 300 µm. **b**–**d** Quantifications of area with plaque load and activated glial cells in deltaE9 and deltaE9/Bace +/− mice at the age of 6–7 months

**Fig. 3:**
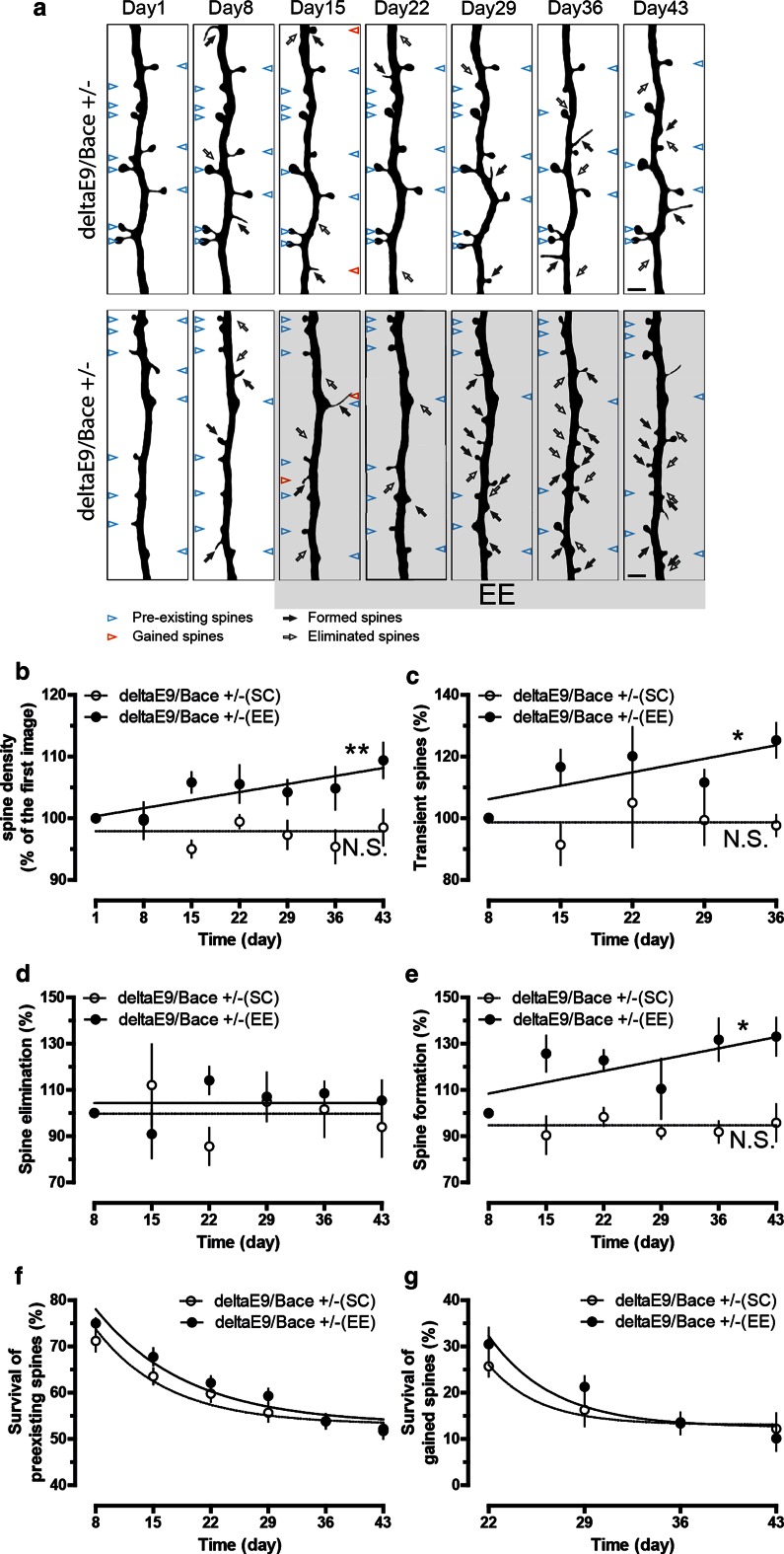
Reduction of BACE1 restores the spine density increase, but not neural circuit remodeling, upon EE in deltaE9 mice. **a** Two-photon micrographs of GFP-labeled apical dendrites. DeltaE9/Bace +/− mice were housed under SC (*above*) or EE (*below*). *Empty or dark arrows* point to eliminated or formed spines compared to previous imaging session. *Blue arrowheads* mark spines that existed in the first imaging session and were stable over the entire imaging period, whereas *red arrowheads* represent gained spines in the first week of EE or matching period of SC that survived over the rest of imaging period. **b**–**e** Quantifications of relative spine density, fraction of transient, eliminated or formed spines. **f**, **g** Fraction of spines in the first imaging session or gained spines in the first week of EE and matching week of SC that survived over the imaging period. *Scale bar* 2 µm

Full-length APP is processed to yield amyloid beta, the principal component of amyloid plaques, through sequential enzymatic cleavage by β- and γ-secretases. To investigate if elevated amyloid beta levels contribute to the impaired adaptive spine plasticity in deltaE9 mice, we crossed deltaE9 mice with BACE1 knockout mice to obtain deltaE9 genotype containing a heterozygous BACE1 gene knockout (deltaE9/Bace +/−). BACE1 is the primary β-secretase. Of note, the density and dynamics of dendritic spines in deltaE9/Bace +/− genotype remained unchanged compared to control or deltaE9 mice, when they were housed under SC (Suppl. Fig. 3b–d). Partial reduction of BACE1 activity dramatically reduced amyloid plaques, glial cell activation and amyloid pathology (Fig. 2, Suppl. Fig. 4 and Suppl. Fig. 5). Unlike deltaE9 group, deltaE9/Bace +/− mice gained the adaptive increase in spine density housed under EE (Fig. 3a, b). To our surprise, the increase in spine density was caused by boosting spine formation (Fig. 3e) instead of decreasing spine elimination (Fig. 3d), which was opposite to the observations in the control group (Fig. 1d, e). In addition, the fates of spines that existed before or were newly formed after EE were indistinguishable between different housing conditions (Fig. 3f, g). An increased fraction of transient spines (Fig. 3c) suggested that the newly gained spines induced by EE were unstable over 1 week. In deltaE9 or control mice, the increased transient fraction during EE was not observed (data not shown). Taken together, EE induced spine density increase but failed to remodel neural circuits in deltaE9/Bace +/− mice. These deficits in neural network remodeling appear to be caused by the reduction of β-secretase, as similar findings have been observed in Bace +/− mice (Suppl. Figure 6). The restoration of adaptive spine density increase suggests removal of amyloid plaques might ameliorate the impaired adaptive plasticity of dendritic spines in preclinical AD.

### Pioglitazone rescues the deficits of adaptive dendritic spine plasticity in deltaE9 mice

**Fig. 4:**
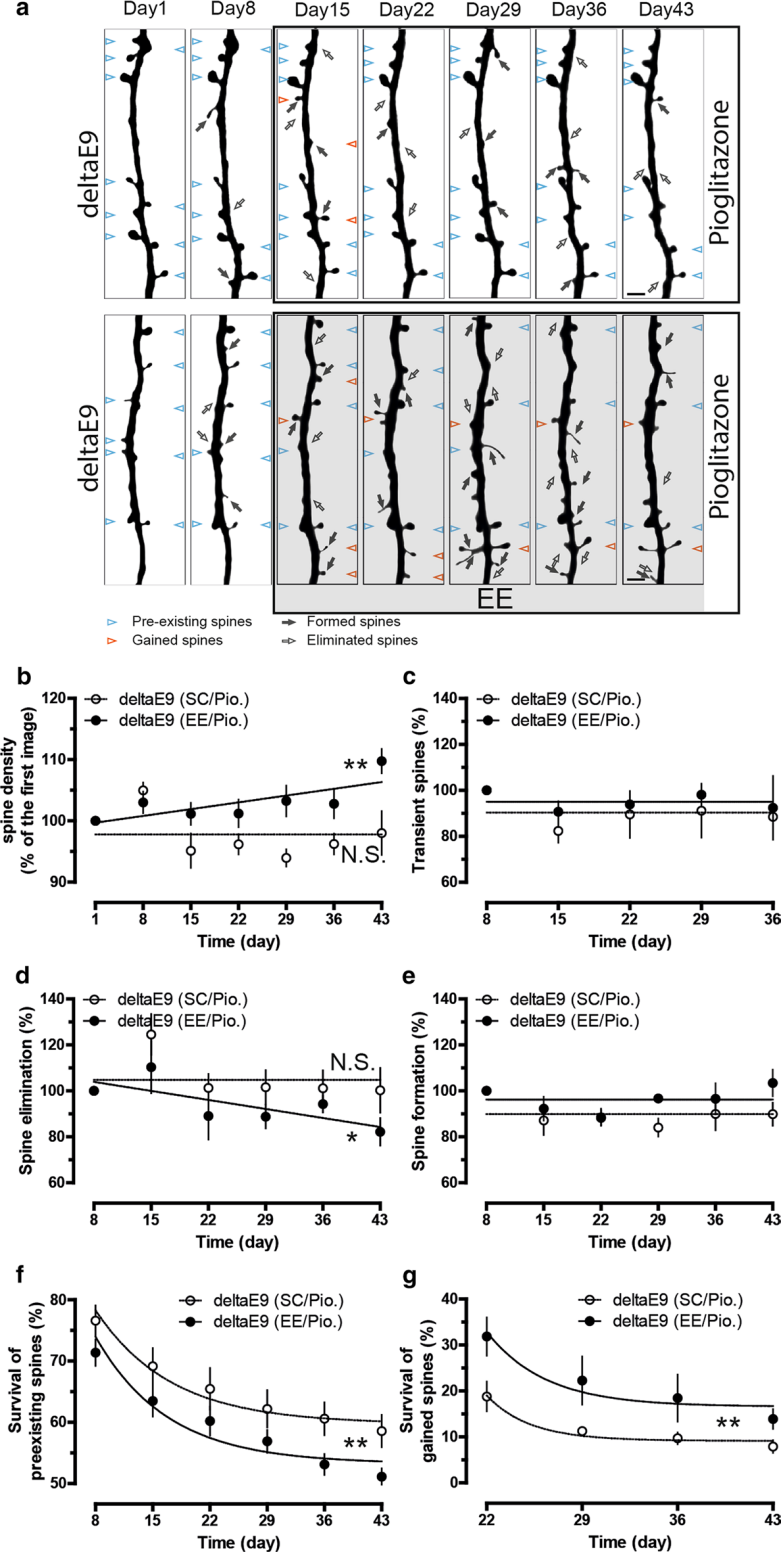
Pioglitazone recovers the observed impairments of spine structural plasticity in deltaE9 mice. **a** Two-photon micrographs of GFP-labeled apical dendrites. DeltaE9 mice were fed with pioglitazone during EE or a matching period of SC. *Empty or dark arrows* point to eliminated or formed spines compared to previous imaging session. *Blue arrowheads* mark spines that existed in the first imaging session and were stable over the entire imaging period while *red arrowheads* represent gained spines in the first week of EE or matching period of SC that survived over the rest of imaging period. **b**–**e** Quantifications of relative spine density, fraction of transient, eliminated or formed spines. **f**, **g** Fraction of spines in the first imaging session or gained spines in the first week of EE and matching week of SC that survived over the imaging period. *Scale bar* 2 µm

As the imaged dendrites were located in amyloid plaque-free brain regions [58], it was plausible to hypothesize that diffusible factors originating from amyloid deposits might contribute to the lack of response of spine density upon EE, which was restored by the removal of plaques (Fig. 3b). Particularly, it is known that amyloid plaques are surrounded by activated glial cells that are known to release pro-inflammatory cytokines [53]. To investigate if these cytokines caused the impaired adaptive plasticity, we treated deltaE9 mice with pioglitazone, a PPAR-gamma agonist, which inhibits the production of pro-inflammatory cytokines without affecting synaptic plasticity [6, 25] (Suppl. Figure 7). Pioglitazone treatment successfully rehabilitated the steady increase of spine density in deltaE9 mice during exposure to EE (Fig. 4a, b). Like in control mice, the EE-induced spine density increase resulted from the gradual decline in spine elimination, while the rate of spine formation was unchanged (Fig. 4d, e). Moreover, less preexisting spines and more gained spines were observed during EE, when deltaE9 mice were fed with pioglitazone (Fig. 4f, g). The fraction of transient spines also remained unchanged (Fig. 4c). These results indicate that the failure of remodeling neural networks upon EE in deltaE9 mice is ascribed to the up-regulation of pro-inflammatory cytokines.

### IL-1 RA rehabilitates the impaired adaptive plasticity of dendritic spines in deltaE9 mice

**Fig. 5:**
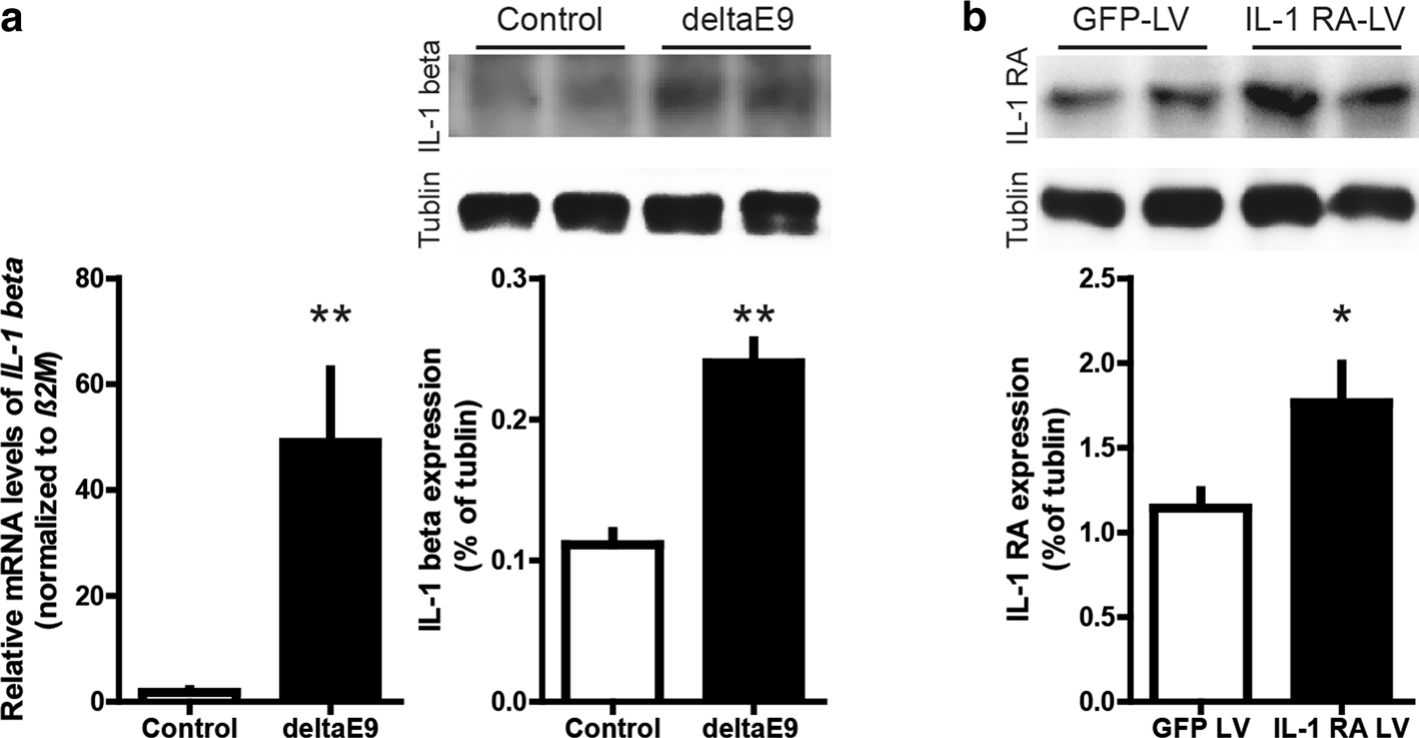
Elevated expression of interleukin-1β in deltaE9 mice and exogenous interleukin-1 receptor antagonist produced by lentivirus infection. **a** By quantitative real-time PCR (*left*) and western blot (*right*), the expression of interleukin-1β (IL-1β) was found to be increased in deltaE9 mice. **b** Interleukin-1 receptor antagonist (IL-1 RA) was overexpressed in somatosensory cortex after 1 month of lentivirus injection, as illustrated by western blot images and quantification

**Fig. 6:**
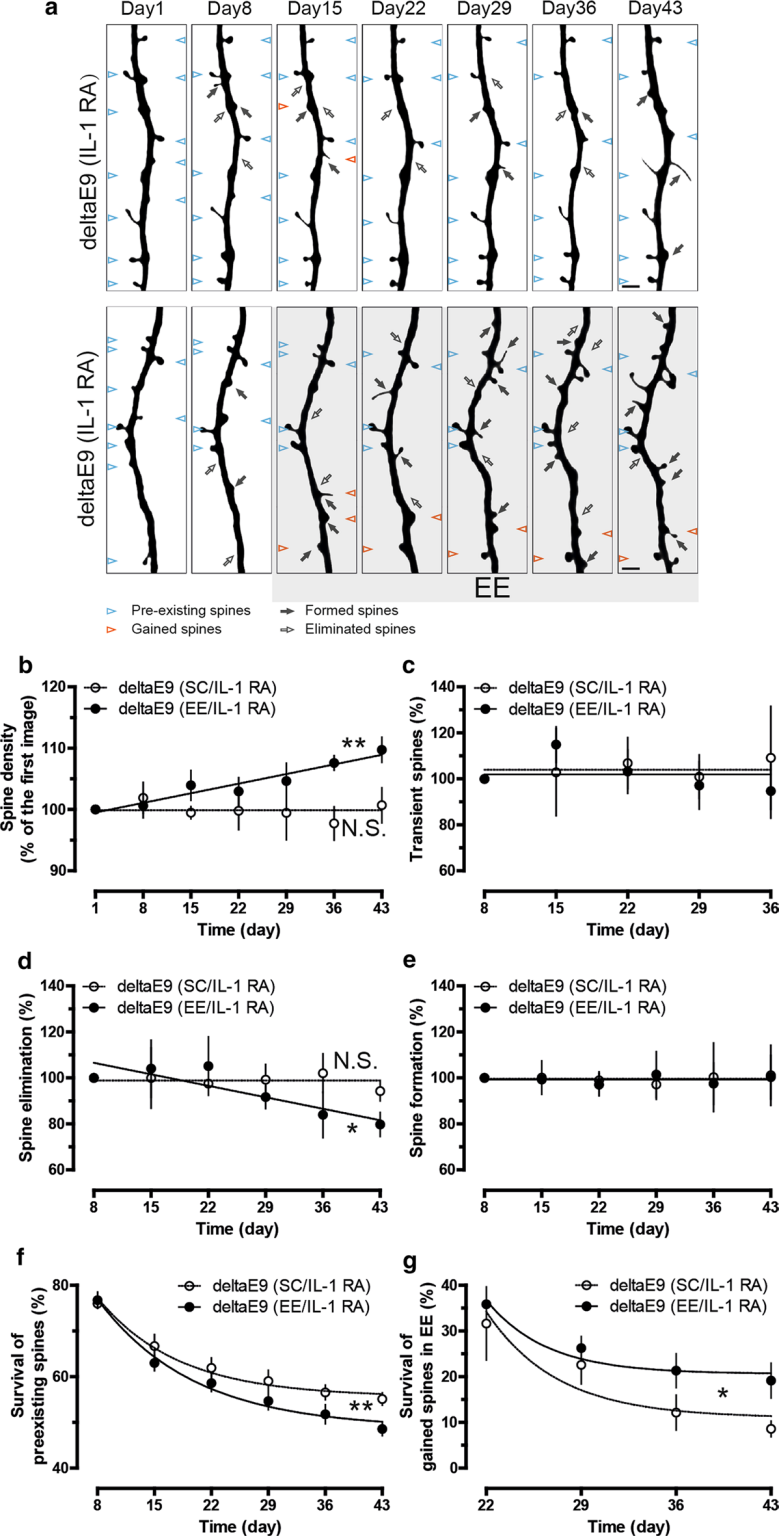
IL-1 RA rescues the impaired adaptive plasticity of dendritic spines in deltaE9 mice. **a** Two-photon micrographs of GFP-labeled apical dendrites of layer V pyramidal neurons. Mice were housed in SC (*above*) or EE (*below*). *Empty or dark arrows* point to eliminated or formed spines compared to previous imaging session. *Blue arrowheads* mark spines that existed in the first imaging session and were stable over the entire imaging period while *red arrowheads* represent gained spines in the first week of EE or matching period of SC that survived over the rest of imaging period. **b**–**e** Quantifications of relative spine density, fraction of transient, eliminated or formed spines. **f**, **g** Fraction of spines in the first imaging session or gained spines in the first week of EE and matching week of SC that survived over the imaging period. *Scale bar, * 2 µm.

The known deleterious effects of interleukin-1β (IL-1β), a key mediator of the inflammatory response in AD, on synaptic plasticity [48] prompted us to examine whether up-regulated levels of IL-1β undermined the adaptive spine plasticity. Consistent with previous reports [33, 47, 55], the expression of IL-1β was indeed significantly enhanced in deltaE9 mice (Fig. 5a). To diminish IL-1β activity, we injected lentivirus (LV) expressing interleukin-1 receptor antagonist (IL-1 RA) [49] into the somatosensory cortex (Fig. 5b). IL-1 RA has previously been found not to alter spine density, LTP, spatial memory and synaptic markers in wild-type mice [9]. In this study, we found that IL-1 RA rectified the adaptive gain of spines upon EE in deltaE9 mice, accompanied with the gradual decline in spine elimination instead of rising spine formation (Fig. 6a, b, d, e). Also, the fate of spines that existed before or newly formed during EE was normalized in deltaE9 mice administered with IL-1 RA (Fig. 6f, g). In addition, the fraction of transient spines was unchanged (Fig. 6c). Taken together, these data suggest up-regulated IL-1β perturbs EE-induced reorganization of neural networks.

## Discussion

As excitatory postsynaptic compartments, dendritic spines receive and integrate informational input from presynaptic terminals [57]. This function is supposed to be disturbed at the very early stages of AD pathogenesis, which may explain why synaptic loss is a much better indicator for cognitive impairment in AD than Aβ burden or neuronal loss [41]. With the advent of cognitive decline, irreversible damage may have already occurred. Prevention strategies in the asymptomatic stages of AD are therefore warranted.

Preclinical AD is replicated in young deltaE9 mice that develop amyloid deposits before the onset of cognitive decline [4, 14, 27, 34, 50]. In this study, we found that 4–5-month-old deltaE9 mice did not increase dendritic spine density when housed under EE in contrast to control mice. The novel external environment also failed to remodel neural networks in these transgenic mice. Reduction of BACE1 activity in deltaE9 mice reduced the deposition of Aβ and restored the increase of spine density during EE, but not the impaired reorganization of neural networks. However, anti-inflammatory treatments, pioglitazone and IL-1 RA, successfully rescued the spine density increase and neural network remodeling upon EE in deltaE9 mice. These results suggest that neuroinflammation contributes to impaired adaptive plasticity of dendritic spines in preclinical stages of AD.

Structural plasticity of dendritic spines refers to the change of their distribution in response to experience [12]. Learning and sensory experience have been reported to remodel neural connections through de novo growth and loss of dendritic spines, which provides a structural substrate for adaptive behaviors. Spine density increases after spatial learning tasks or manipulations that intensify sensory inputs [26, 28], while deprivation of sensory experience leads to a decrease in spine density [52]. This structural synaptic plasticity may substantially boost information storage capacity in the brain [7]. The failure to increase spine density in young adult deltaE9 mice upon EE suggests an impairment of experience-dependent structural plasticity in asymptomatic stages of AD, before spine loss occurs. In addition, stabilized newly formed spines and destabilized preexisting spines in novel experience reflect a rewiring of neural networks, which facilitates a quicker adaption of brain to the same situation in the future [19, 54, 56]. Interestingly, the ability to dismantle the preexisting neural networks in novel external environment remains intact in deltaE9 mice. However, deltaE9 mice fall short of the establishment of novel neural networks. These results imply that experience-dependent demolition and construction of neural networks are two processes that are independent from each other.

BACE1 initiates the proteolytic process of APP into Aβ, which accumulates to form amyloid plaques. As Aβ is believed to play a central role in AD, BACE1 becomes an attractive drug target. Indeed, partial reduction of BACE1 activity leads to dramatic reductions on amyloid plaque burden and synaptic deficits with a small decrease of Aβ levels in young AD transgenic mice [29]. However, pharmacological inhibition of BACE1 impairs structural and functional synaptic plasticity implying its physiological role in dendritic spines [11]. The boosted transient spines, which contribute to increased spine formation, in deltaE9/Bace +/− mice during EE indicate the maintenance of experience-dependent synaptic rearrangement requires physiological level of BACE1 activity. It still remains unclear, whether BACE1 itself or its substrates are involved in synaptic physiology.

Amyloid deposition, driving neuroinflammation, is associated with activated glial cells and the release of pro-inflammatory cytokines. These soluble mediators, IL-1β in particular, directly and extensively disturb synaptic transmission and plasticity. IL-1β regulates the expression and phosphorylation of glutamate receptors on dendritic spines [36]. The altered sensitivity of receptors to synaptic glutamate modulates synaptic plasticity. In addition, IL-1β disrupts BDNF signaling cascades and thereby prevents activity-driven formation of filamentous actin in spines, which is required for spine structural plasticity [48]. The restorative effects of pioglitazone and IL-1 RA demonstrated herein implicate a deleterious role of IL-1β in experience-dependent spine structural plasticity preceding cognitive impairment in AD.

Of note, numerous clinical studies have demonstrated that anti-inflammatory treatment reduces dementia risk or delay the onset of AD [3, 8, 18], although anti-inflammatory drugs in clinically manifested AD failed to be proven effective [15, 24]. These trials suggest prevention of inflammatory processes is beneficial at the preclinical stages of AD. Our data confirm that neuroinflammation caused impairments of spine structural plasticity is curable by anti-inflammatory treatment in a preclinical mouse model of AD. This finding implies the normalization of adaptive structural plasticity of dendritic spines may correlate with the beneficial effects of anti-inflammatory treatment in preclinical AD patients.

We conclude that our in vivo dendritic spine analysis reveals that neuroinflammation, caused by amyloid deposition, undermines the adaptive changes of neural networks upon novel external environment before the occurrence of dementia, providing new insights for a possible benefit of anti-inflammatory treatments in preclinical AD.

## Electronic supplementary material

Supplementary material 1 (TIFF 2978 kb) Supplementary Fig. 1. Transcranial in vivo two-photon imaging and housing conditions. (**a**) Transcranial in vivo two-photon imaging was taken in somatosensory cortex (left, black circle). Lateral view of GFP-labeled layer V pyramidal cortical neurons is in the middle. Apical tuft dendrites of layer V neurons were imaged at 20-70 µm depths (right). Scale bar represents 100 µm. (**b**) Schematic drawing of an EE cage (left) and a cage of SC (right).

Supplementary material 2 (TIFF 5509 kb) Supplementary Fig. 2. The elimination rate of newly gained dendritic spines induced by EE is higher in deltaE9 mice. (**a, b**) Fractions of spines from the first imaging session that remained stable during the whole imaging period when mice housed under SC or EE. (**c, d**) Fractions of newly gained spines in the first week of EE or matching period of SC that remained stable during the whole imaging period in control and deltaE9 mice.

Supplementary material 3 (TIFF 3747 kb) Supplementary Fig. 3. Partial reduction of BACE1 in deltaE9 mice does not change spine density and dynamics. (**a-c**) Quantifications of spine density, fraction of eliminated or formed spines in mice at the age of 4-5 months housed under SC (calculated from dendrites collected in day1 and day8 that were demonstrated in Fig. 1 and Fig. 3).

Supplementary material 4 (TIFF 2766 kb) Supplementary Fig. 4. Images of amyloid plaques stained by methoxy-X04 and activated glial cells with higher resolution. Immunohistochemical labeling of amyloid plaques (blue), activated astrocytes (GFAP, red) and microglia (Iba-1, red) in the cortex of deltaE9 mice. Scale bar = 20 µm.

Supplementary material 5 (TIFF 7097 kb) Supplementary Fig. 5. Amyloid pathology in deltaE9/Bace +/− mice is reduced. (**a**) Immunohistochemical labeling of amyloid deposits by methoxy-X04 (blue) and 4G8 (red) in the cortex. Scale bar = 100 µm. (**b-d**) Quantifications of area with methoxy or 4G8 staining in transgenic mice at the age of 4-5 months (4 M) or 6-7 months (6 M).

Supplementary material 6 (TIFF 15894 kb) Supplementary Fig. 6. Reduction of BACE1 impairs neural circuit remodeling upon EE. (**a**) Two-photon micrographs of GFP-labeled apical dendrites. Bace +/− mice were housed under SC (above) or EE (below). Empty or dark arrows point to eliminated or formed spines compared to previous imaging session. Blue arrowheads mark spines that existed in the first imaging session and were stable over the entire imaging period, whereas red arrowheads represent gained spines in the first week of EE or matching period of SC that survived over the rest of imaging period. (**b-e**) Quantifications of relative spine density, fraction of transient, eliminated or formed spines. (**f, g**) Fraction of spines in the first imaging session or gained spines in the first week of EE and matching week of SC that survived over the imaging period. Scale bar = 2 µm.

Supplementary material 7 (TIFF 15850 kb) Supplementary Fig. 7. Structural plasticity of dendritic spines in control mice with pioglitazone treatment. (**a**) Two-photon micrographs of GFP-labeled apical dendrites. Control mice were fed with pioglitazone. Empty or dark arrows point to eliminated or formed spines compared to previous imaging session. Blue arrowheads mark spines that existed in the first imaging session and were stable over the entire imaging period, whereas red arrowheads represent gained spines in the first week of EE or matching period of SC that survived over the rest of imaging period. (**b-e**) Quantifications of relative spine density, fraction of transient, eliminated or formed spines. (**f, g**) Fraction of spines in the first imaging session or gained spines in the first week of EE and matching week of SC that survived over the imaging period. Scale bar = 2 µm.
