# Supplementary figures and images for: Neuroinflammation impairs adaptive structural plasticity of dendritic spines in a preclinical model of Alzheimer’s disease
Source: Acta Neuropathol. 2016 Jan 2;131:235–46. doi: 10.1007/s00401-015-1527-8 (PMC4713725; doi:10.1007/s00401-015-1527-8)

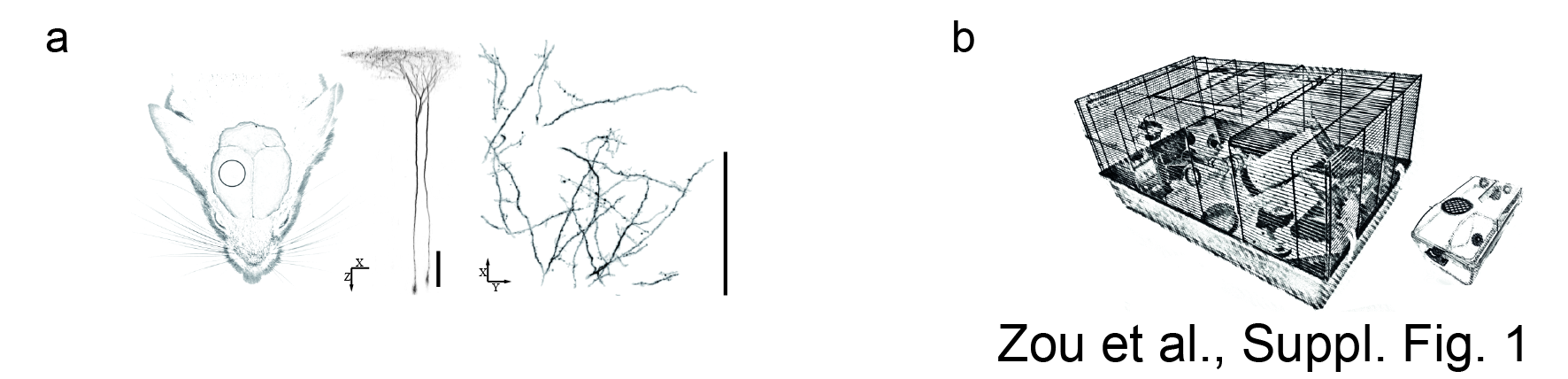

Supplement: Supplementary file 1 — Supplementary material 1 (TIFF 2978 kb) Supplementary Fig. 1. Transcranial in vivo two-photon imaging and housing conditions. (a) Transcranial in vivo two-photon imaging was taken in somatosensory cortex (left, black circle). Lateral view of GFP-labeled layer V pyramidal cortical neurons is in the middle. Apical tuft dendrites of layer V neurons were imaged at 20-70 µm depths (right). Scale bar represents 100 µm. (b) Schematic drawing of an EE cage (left) and a cage of SC (right). [file 401_2015_1527_MOESM1_ESM.tif]

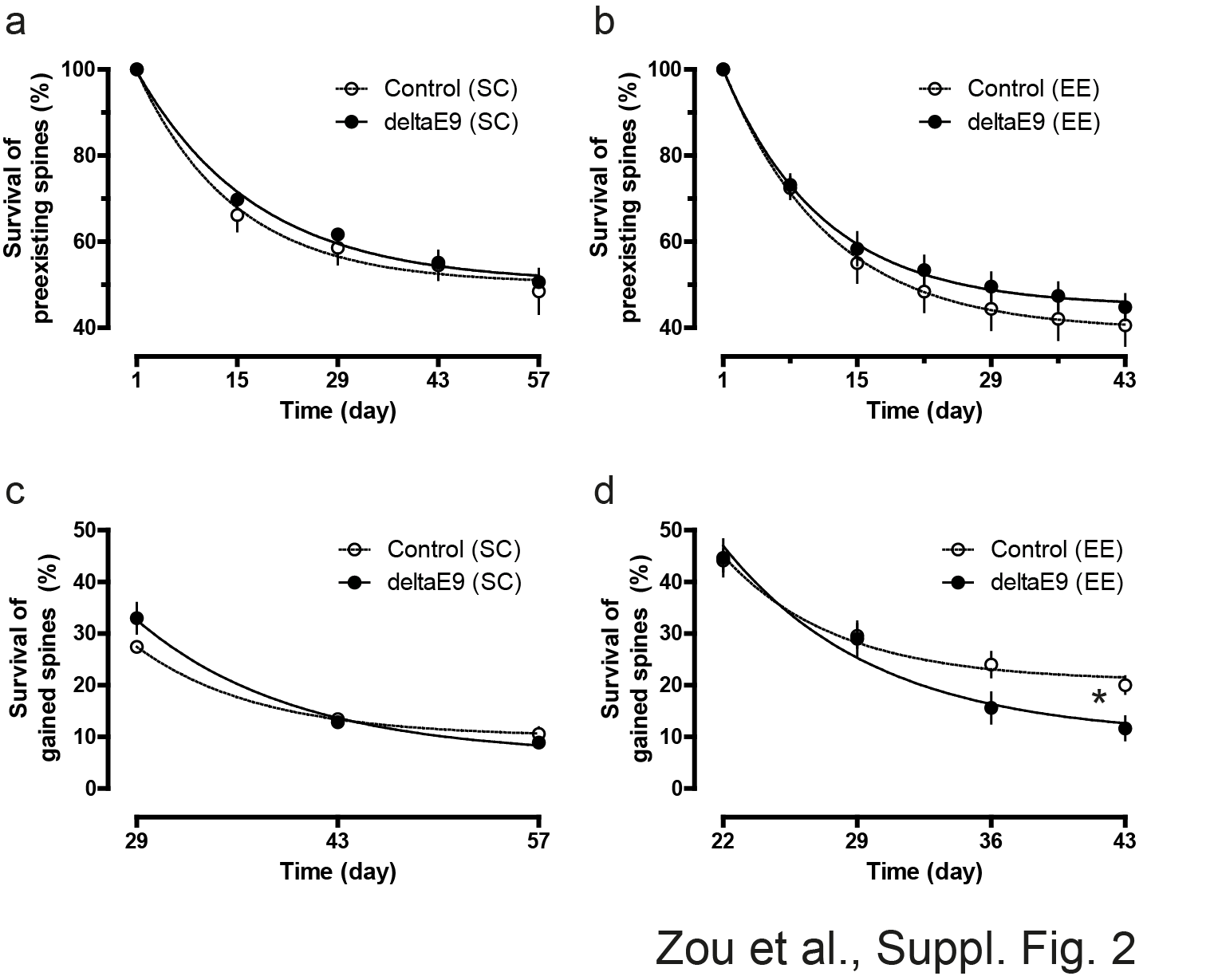

Supplement: Supplementary file 2 — Supplementary material 2 (TIFF 5509 kb) Supplementary Fig. 2. The elimination rate of newly gained dendritic spines induced by EE is higher in deltaE9 mice. (a, b) Fractions of spines from the first imaging session that remained stable during the whole imaging period when mice housed under SC or EE. (c, d) Fractions of newly gained spines in the first week of EE or matching period of SC that remained stable during the whole imaging period in control and deltaE9 mice. [file 401_2015_1527_MOESM2_ESM.tif]

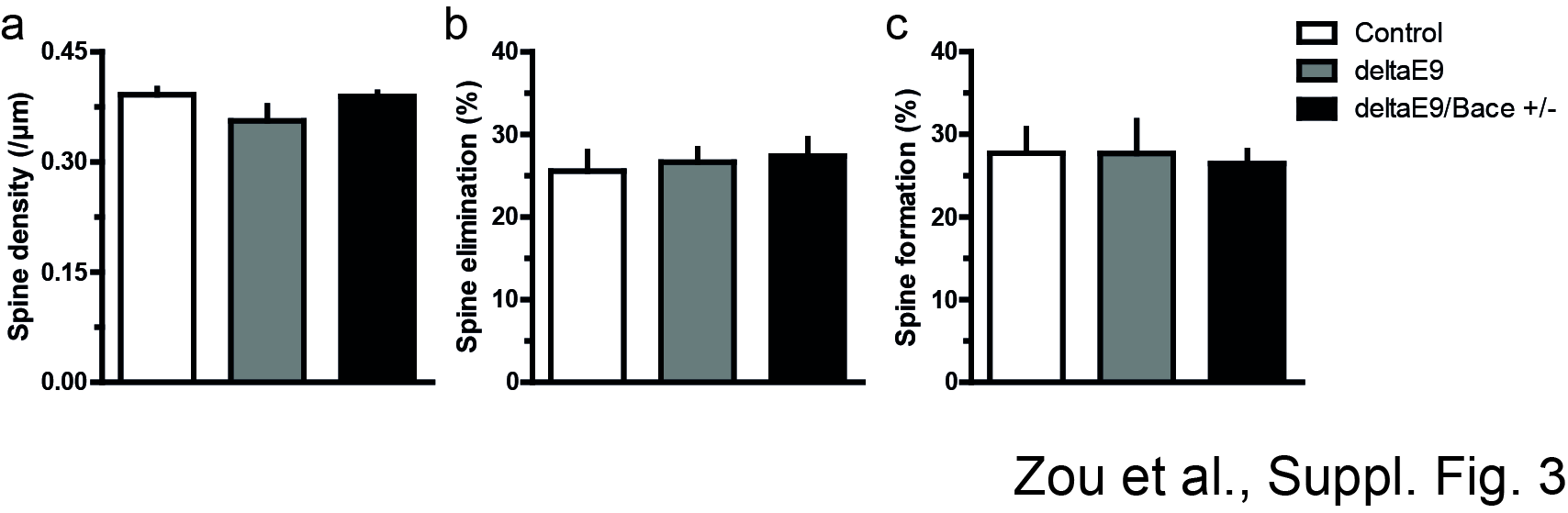

Supplement: Supplementary file 3 — Supplementary material 3 (TIFF 3747 kb) Supplementary Fig. 3. Partial reduction of BACE1 in deltaE9 mice does not change spine density and dynamics. (a-c) Quantifications of spine density, fraction of eliminated or formed spines in mice at the age of 4-5 months housed under SC (calculated from dendrites collected in day1 and day8 that were demonstrated in Fig. 1 and Fig. 3). [file 401_2015_1527_MOESM3_ESM.tif]

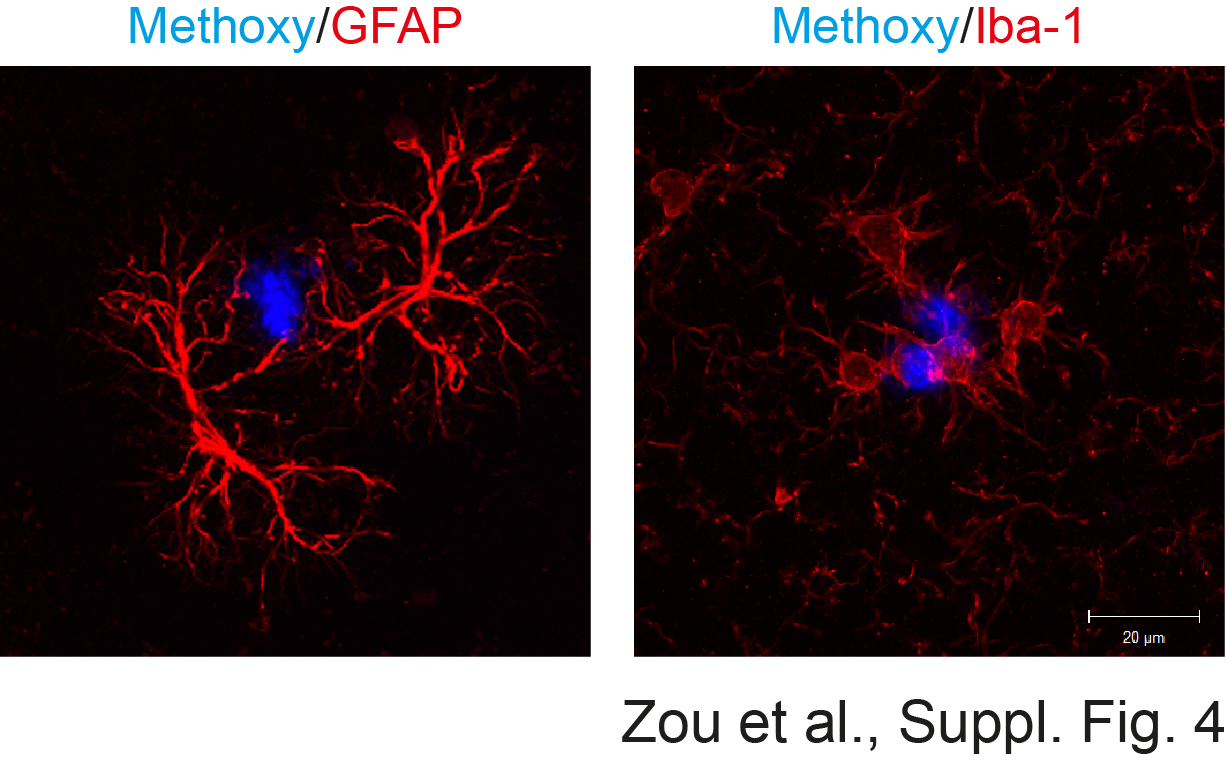

Supplement: Supplementary file 4 — Supplementary material 4 (TIFF 2766 kb) Supplementary Fig. 4. Images of amyloid plaques stained by methoxy-X04 and activated glial cells with higher resolution. Immunohistochemical labeling of amyloid plaques (blue), activated astrocytes (GFAP, red) and microglia (Iba-1, red) in the cortex of deltaE9 mice. Scale bar = 20 µm. [file 401_2015_1527_MOESM4_ESM.tif]

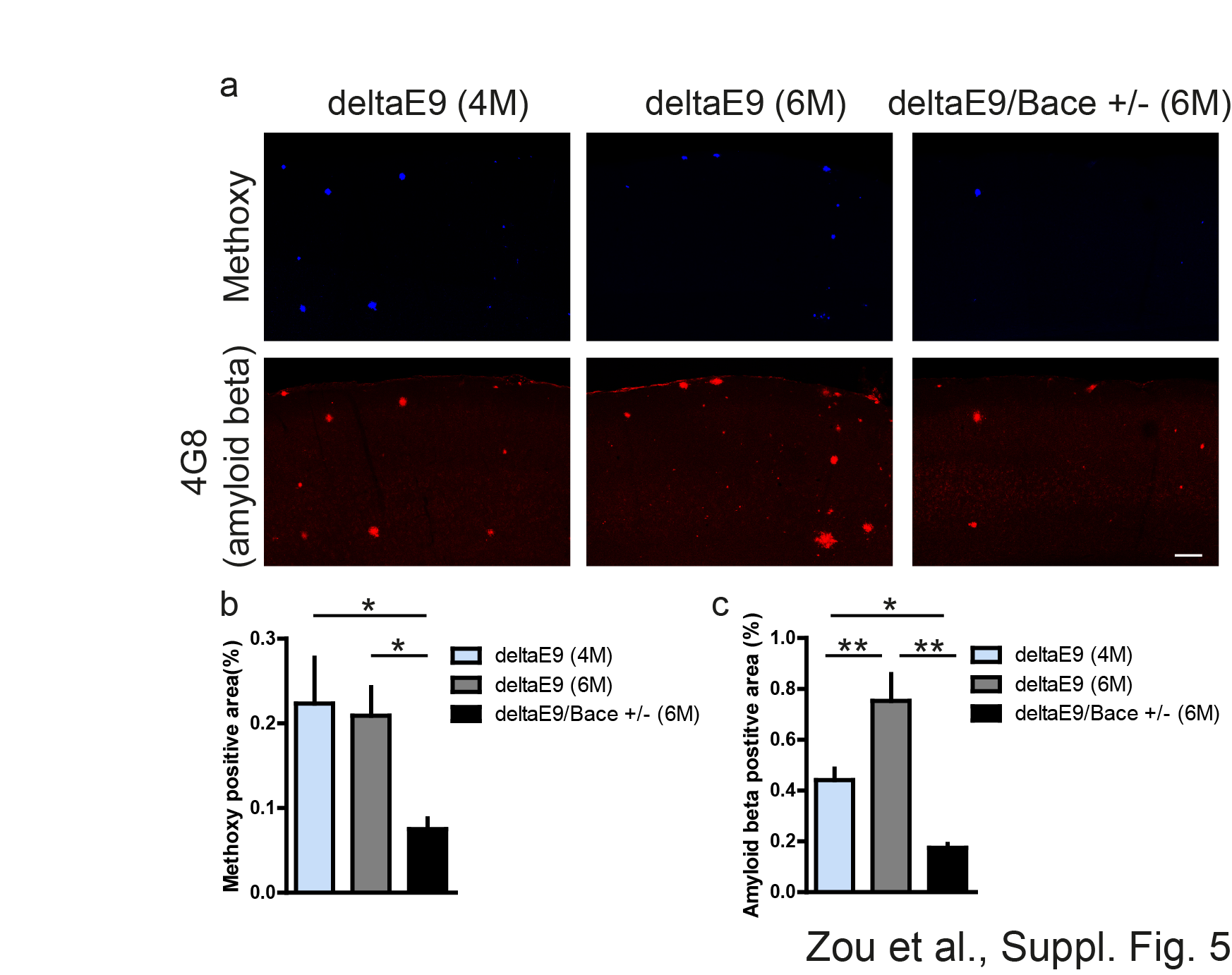

Supplement: Supplementary file 5 — Supplementary material 5 (TIFF 7097 kb) Supplementary Fig. 5. Amyloid pathology in deltaE9/Bace +/− mice is reduced. (a) Immunohistochemical labeling of amyloid deposits by methoxy-X04 (blue) and 4G8 (red) in the cortex. Scale bar = 100 µm. (b-d) Quantifications of area with methoxy or 4G8 staining in transgenic mice at the age of 4-5 months (4 M) or 6-7 months (6 M). [file 401_2015_1527_MOESM5_ESM.tif]

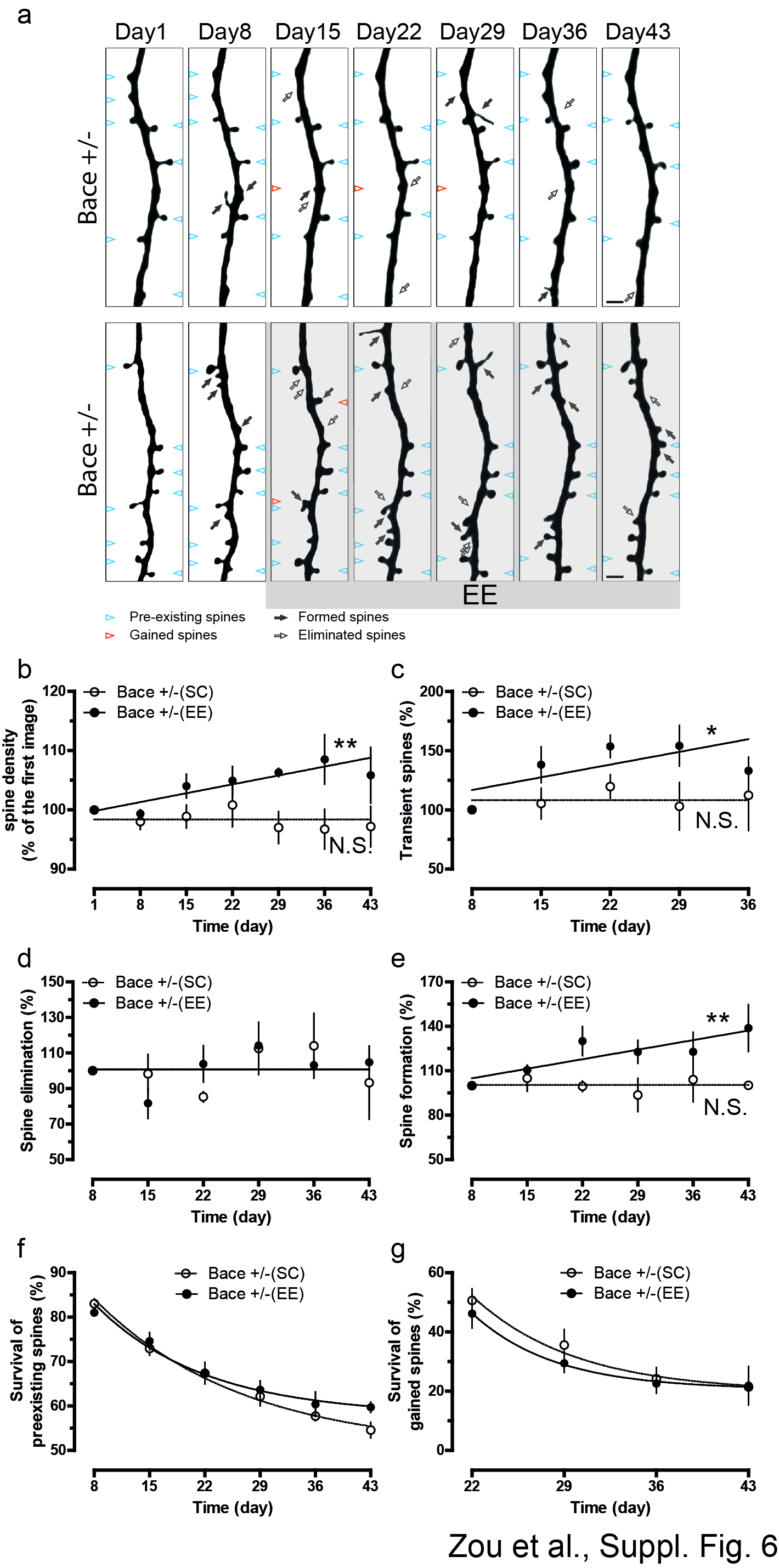

Supplement: Supplementary file 6 — Supplementary material 6 (TIFF 15894 kb) Supplementary Fig. 6. Reduction of BACE1 impairs neural circuit remodeling upon EE. (a) Two-photon micrographs of GFP-labeled apical dendrites. Bace +/− mice were housed under SC (above) or EE (below). Empty or dark arrows point to eliminated or formed spines compared to previous imaging session. Blue arrowheads mark spines that existed in the first imaging session and were stable over the entire imaging period, whereas red arrowheads represent gained spines in the first week of EE or matching period of SC that survived over the rest of imaging period. (b-e) Quantifications of relative spine density, fraction of transient, eliminated or formed spines. (f, g) Fraction of spines in the first imaging session or gained spines in the first week of EE and matching week of SC that survived over the imaging period. Scale bar = 2 µm. [file 401_2015_1527_MOESM6_ESM.tif]

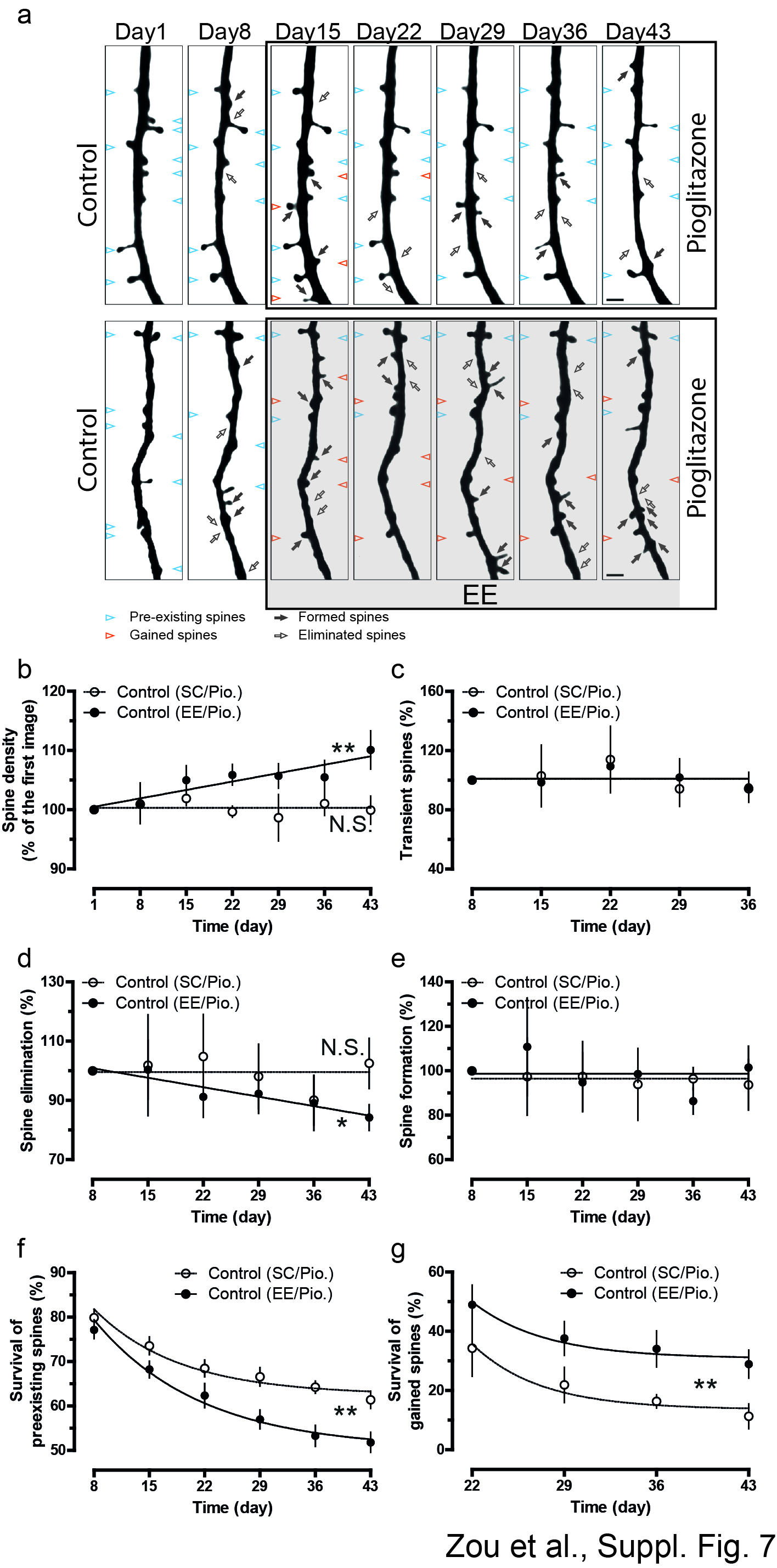

Supplement: Supplementary file 7 — Supplementary material 7 (TIFF 15850 kb) Supplementary Fig. 7. Structural plasticity of dendritic spines in control mice with pioglitazone treatment. (a) Two-photon micrographs of GFP-labeled apical dendrites. Control mice were fed with pioglitazone. Empty or dark arrows point to eliminated or formed spines compared to previous imaging session. Blue arrowheads mark spines that existed in the first imaging session and were stable over the entire imaging period, whereas red arrowheads represent gained spines in the first week of EE or matching period of SC that survived over the rest of imaging period. (b-e) Quantifications of relative spine density, fraction of transient, eliminated or formed spines. (f, g) Fraction of spines in the first imaging session or gained spines in the first week of EE and matching week of SC that survived over the imaging period. Scale bar = 2 µm. [file 401_2015_1527_MOESM7_ESM.tif]
